# Effectiveness of Nutrition Education on Weight Loss and Body Metrics Among Obese Adults: An Interventional Study

**DOI:** 10.7759/cureus.74373

**Published:** 2024-11-24

**Authors:** Anisa Faiz, Sadia Nawaz, Qaisar Raza, Kinza Imran, Rakhshanda Batool, Sehrish Firyal, Shahana Bashar, Maleeha Imran

**Affiliations:** 1 Food Science and Human Nutrition, University of Veterinary and Animal Sciences, Lahore, PAK; 2 Biochemistry and Biotechnology, University of Veterinary and Animal Sciences, Lahore, PAK; 3 Health Sciences, College of Applied Health Sciences, A’Sharqiya University, Ibra, OMN; 4 Pharmaceutical Sciences, University of Veterinary and Animal Sciences, Lahore, PAK

**Keywords:** adults, intervention, lahore, nutrition education, nutrition education intervention, obesity, weight loss

## Abstract

Background

Obesity has detrimental personal, societal, and economic consequences and raises the risk of developing chronic diseases such as diabetes and cardiovascular diseases. Diet and exercise behaviors are frequently the focus of weight loss programs. Effective nutrition education is associated with a reduced risk of chronic diseases and body weight management. Individualized lifestyle and counseling sessions with follow-ups reduce weight loss compared to single combined sessions*.*

Objectives

The objective of this study was to assess the effectiveness of nutrition education intervention on weight loss among adults aged 18-40 years in Lahore through an interventional study.

Methods

This study was conducted in Lahore. According to the inclusion criteria, overweight/obese (Body Mass Index (BMI) ≥ 23 kg/m²), young (18-40 years old) men and women were part of this study. A total of 50 participants were randomized into two groups, one as the control group and the other one as the intervention group. Group counseling was provided to the control group. The intervention group was assessed individually through follow-ups for 3 months of online and on-campus sessions. Data was collected at four intervals at baseline, month 1, month 2, and month 3 through a self-administered assessment form. Data was analyzed using the statistical package for social science, SPSS version 25 (IBM Corp., Armonk, USA).

Results

Out of the 50 participants, 54% (27) were women while 46% (23) were men (mean age 29.90, SD = 6.26, BMI at baseline 32.18 kg/m², 26. 49 kg/m²), and randomized by a blinded researcher. At three months, the mean differences in BMI, waist circumference, waist-to-hip ratio, and body fat percentage between the groups were 3 kg (23.44 ± 0.58, 95% CI 22.86-24.02), 33.90 cm (95% CI 32.50-35.30), 0.86 (95% CI 0.74-0.98), and 24.79% (95% CI 18.00-31.58), respectively, in favor of the intervention group (p < 0.005).

Conclusion

This study demonstrates that a structured dietary intervention combined with physical activity guidance leads to significant weight loss and improved body composition in obese adults. These findings support the effectiveness of comprehensive weight loss strategies for managing obesity.

## Introduction

Obesity is a global public health problem. In 2030, it is expected that 1.12 billion individuals worldwide will meet the criterion for obesity, reflecting a sharp increase in the prevalence of obesity [1]. World Health Organization reported that over 1.9 billion adults aged 18 and over were overweight, and over 650 million were obese worldwide in 2016 [2]. Data from the 1998 National Health Interview Survey shows that 58% of obese women and 50% of obese men in the US are actively attempting to lose weight. Unfortunately, despite the efforts of a significant percentage of the population, the prevalence of obesity has remained high. Obesity and overweight are the leading causes of morbidity and mortality. Making small lifestyle changes in food and exercise routines may prevent weight gain steadily [3,4].

Excess body weight raises the risk of chronic diseases, although even modest weight loss (3%-5% drop of baseline body weight) can improve health parameters like cholesterol, blood glucose, and blood pressure and reduce the risk of chronic diseases like diabetes and cardiovascular disease. Anti-obesity phytochemicals are found in fruits and vegetables, which limit excessive lipase levels and adipose tissue growth, hence controlling the onset and progression of obesity and maintaining body weight. Fruits and vegetables are very low in energy because they contain high amounts of water and fiber [5]. Choosing a diet that is rich in nutrient-dense fruits and vegetables, whole grains, nuts and seeds, and legumes and lower in trans fats and saturated fat, refined carbohydrates, red meat, sugar-sweetened foods and beverages helps lose weight. These nutrient-dense foods tend to decrease daily calorie consumption and meal portions [6].

People who alternate days of fasting with days of feasting, during which they are allowed to eat whatever they want, tend to lose 3%-7% of their body weight over the course of two to three months. These people also experience improvements in their lipid profiles, blood pressure, and insulin sensitivity, which makes them more likely to stick to their diet and lose weight. As opposed to this, daily calorie restriction among participants drops after a month and keeps declining after that [7]. There is evidence to suggest a persistent, involuntary gain in the body of 0.24 to 0.45 kg per year for women and 0.25 to 0.58 kg per year for men among adults [8].

The Mindfulness-Based Eating Awareness program improves self-regulation of eating triggers such as physical hunger, stomach fullness, taste pleasure, food cravings, emotions, and other eating triggers while calorie intake is restricted [9]. According to a randomized controlled trial (RCT), weight-loss interventions can decrease the risk of obesity. At BMIs of 20 to 25, the risk of early death was at its lowest [10]. Numerous psychological and social effects of obesity are also possible. Lifestyle intervention programs, which include food, physical exercise, and behavioral therapy, are one of the most effective methods for managing obesity. Treatment efficiency, health outcomes, and the overall burden of chronic disease will all improve with increased adherence to lifestyle intervention programs and health guidelines among the general population [11]. People with lower socioeconomic status (SES) are more likely to be obese than those with higher SES. According to a study, patients who are from more deprived areas were more likely to undergo obesity treatment [12].

The tremendous consequences of being overweight and obese on the social, economic, and individual levels highlight the urgent need for successful weight control programs. Numerous weight-management strategies have been developed to change eating and exercise habits [13]. The most frequently reported weight-loss approaches, according to the National Health Interview Study, are calorie restriction alone, eating less fat, and exercising more. The authors said that individuals who lost more weight before beginning the programs were more likely to sustain their weight reduction, and those who exercised more were able to maintain their weight loss better than those who did not. Participants will not lose weight if they are unable to stick to weight loss programs [14]. 

A randomized controlled trial was conducted in the USA to examine whether vegetarian diets may promote weight loss. This trial included 1151 participants who received the intervention for a median of 18 weeks. Results showed that individuals who were assigned to vegetarian diet groups lost considerably more weight than those assigned to non-vegetarian diet groups (weighted mean difference, 2.02 kg; 95% CI: 2.80-1.23) [15]. Obesity poses adverse effects on the health of adults, quality of life, and healthcare costs. Moreover, there is a lack of research among adults in Lahore regarding nutritional educational intervention.

Therefore, the purpose of this study was to assess the effectiveness of a nutritional educational intervention on weight loss among adults aged 18-40 years in Lahore. Both groups received nutritional education, but the experimental group also received a structured diet plan and physical activity guidelines, while the control group received a single combined counseling session focused on a balanced diet.

## Materials and methods

Study design and population

An interventional study design was used, and data was collected from Lahore, Pakistan. A general population of young adults between the ages of 18 and 40 years from Lahore, Pakistan, were randomly selected for this study.

Study sample

A total of 50 adults residing in Lahore were randomly selected for the study. Individuals, including men and women with a BMI ≥ 23.5 Kg/m^2 ^were selected in this study. According to South Asian cut-off points, a BMI ≤ 18.5 Kg/m^2^ is considered underweight, a BMI of 18.5-23.5 Kg/m^2^ is considered normal weight, and a BMI of 23.5-27 Kg/m^2 ^is considered overweight, while a BMI >27 Kg/m^2 ^comes under the fold of obesity.

Inclusion criteria

Individuals, including men and women between 18 and 40 years of age with a body mass index (BMI) of ≥23.5 Kg/m^2 ^who have previously been sedentary (<60 minutes per week of light activity for the 3 months prior to the study), were included in this study.

Exclusion criteria

People who were under the age of 18 years and older than 40 years of age, having diabetes mellitus and/or following weight-loss diets or taking medications that may affect weight were not included in our study. In addition, patients having previous or planned bariatric surgery, and who were currently participating in a structured and monitored weight-loss program, having eating disorders and/or terminal illness, dementia, a severe mental health problem or learning difficulty, a history of cardiovascular disease, perimenopause or otherwise irregular menstrual cycle, pregnancy, and currently smoking were excluded from our study.

Randomization and intervention groups

The selected adults were randomized into two groups - an intervention group and a control group - for 12 weeks.

Group A (Control Group): Participants in the control group were intervened to maintain their weight through dietary counseling based on a balanced diet provided as a combined session.

Group B (Intervention Group): The intervention group had individualized diet plans and exercise routines. Participants’ fat mass and lean mass were measured every month. Individualized diet plans and exercise guidelines were presented in each month's follow-ups. The dietary component suggested wholesome food options with a focus on modest calorie reduction (typically 500 kcal/day), including a reduction in calorie-dense, nutrient-poor foods, a substitution of simple carbohydrates with whole grains, and an increase in fresh fruit and vegetables, healthy fats, and proteins. The exercise part focused on increasing daily activity and moderate-intensity exercise, most commonly, walking and strength training. The meals followed the American Heart Association guidelines for macronutrient intake, with 30% of energy as fat, 55% as carbohydrate, and 15% as protein. The follow-ups were done through face-to-face interviews and telephonic conversations.

Data collection

A self-administered food frequency questionnaire and diet plans were used as data-collection tools. Our food frequency questionnaire asked about socio-demographic characteristics, anthropometric measurements, lifestyle, dietary assessment, and physical activity-related questions from the participants. A brochure was used as the educational material. The brochure contained information about healthy and unhealthy foods that help in weight loss.

Anthropometric measurements

The following measurements were taken from the participants. To calculate the participant’s BMI, their height, weight, and age were recorded. The waist-to-hip ratio was calculated by taking the waist and hip measurements of the participants. Waist circumference was measured by measuring the waist size. Body fat percentage was calculated by taking the following measurements: neck size, waist and abdomen size, weight in pounds, age, height, and weight**.** All measurements were taken at baseline, month 1, month 2, and month 3.

Statistical analysis

Data was analyzed using SPSS version 25 (IBM Corp., Armonk, USA). Descriptive statistics like mean and percentages were used to describe the socio-demographic variables. Independent t-test was used to compare the differences in groups by assessing the effectiveness of weight-loss interventions. The mean difference in weight change between the intervention and control groups was evaluated.

Ethical approval

Ethical approval was obtained from the ethical review committee of the University of Veterinary and Animal Sciences (UVAS). While conducting the research, rules and guidelines established by the ethical committee of UVAS were adhered to and the rights of the research participants were respected. Verbal consent was obtained from the participants. The gathered data and information were kept private. The participants were also informed that they were free to withdraw anytime during the course of the study.

## Results

Sociodemographic characteristics

Most of the participants were females (54%). In addition, 74% of participants were single while the rest were married. Furthermore, 50% of participants had family earning levels of >50,000 Pakistani Rupee (PKR), while 42% had earning levels between 20,000-50,000 PKR and 8% had income levels of <20,000 PKR. 46% of participants were self-employed; 40% were working in the private sector, 10% were government employees, and 4% were unemployed. The mean age of participants was 25.90 years (Table 1). 

**Table 1:** Socio-demographic characteristics of the participants

Variable		n (%)	
Gender			
Female		27 (54)	
Male		23 (46)	
Marital status			
Single		37 (74)	
Married		13 (26)	
Educational level			
Primary		7 (14)	
Secondary		12 (24)	
Graduation		31 (62)	
Family income (in Pakistani Rupees)			
<20,000		4 (8)	
20-50,000		21 (42)	
>50,000		25 (50)	
Employment status			
Government sector		5 (10)	
Private sector		20 (40)	
Self-employed		23 (46)	
Unemployed		2 (4)	
Age			
Mean:	Std. Error Mean		Std. deviation
25.90 years	.88		6.26

Anthropometric measurements

Body mass index at baseline showed that 58% of the participants were obese, while 42% were overweight at baseline. At the month 1 interval, 64% of the participants were obese and 36% were overweight. At month 2, 78% of the participants were overweight and 22% were obese. In month 3, 56% of participants were overweight, 34% were normal weight, and 10% were obese (Table 2). 

**Table 2:** Anthropometric measurements of adults

Body mass index		n (%)
Baseline	Overweight	21 (42)
	Obesity	29 (58)
Month 1	Overweight	32 (64)
	Obesity	18 (36)
Month 2	Overweight	39 (78)
	Obesity	11 (22)
Month 3	Normal weight	17 (34)
	Overweight	28 (56)
	Obesity	5 (10)
Waist circumference		n (%)
Baseline	Low risk	12 (24)
	High risk	12 (24)
	Very high risk	26 (52)
Month 1	Low risk	15 (30)
	High risk	12 (24)
	Very high risk	23 (46)
Month 2	Low risk	19 (38)
	High risk	24 (48)
	Very high risk	7 (14)
Month 3	Low risk	34 (68)
	High risk	15 (30)
	Very high risk	1 (2)
Body Fat Percentage		n (%)
Baseline	Under fat	1 (2)
	Healthy	10 (20)
	Overfat	14 (28)
	Obese	25 (50)
Month 1	Under fat	1 (2)
	Healthy	10 (20)
	Overfat	17 (34)
	Obese	22 (44)
Month 2	Under fat	1 (2)
	Healthy	17 (34)
	Overfat	18 (36)
	Obese	14 (28)
Month 3	Under fat	1 (2)
	Healthy	28 (56)
	Overfat	18 (36)
	Obese	3 (6)

Waist circumference of participants from baseline to month 3

52% of the participants had very high-risk waist circumference, while 24% had high risk and 24% had low risk at baseline level. In month 1, 46% of the participants were at very high risk, 30% were at low risk, and 24% were at high risk. In month 2, 48% of participants were at high risk, 38% were at low risk, and 14% were at very high risk. In the month 3 interval, 68% of the participants were at low risk, 30% were at high risk, and 2% were at very high risk.

Waist-to-hip ratio of participants from baseline to month 3

At baseline, 40% of the participants had a low-risk waist-to-hip ratio, while 22% had moderate risk and 38% had very high risk. In month 1, 46% of the participants had a low-risk waist-to-hip ratio, while 34% had moderate risk and 20% had high risk. In month 2, 50% of the participants had a low-risk waist-to-hip ratio, while 46% had moderate risk and 10% had high risk. In month 3 interval, 68% of the participants had low risk, while 32% had moderate risk.

Body fat percentage of participants from baseline to month 3

At baseline, 50% of the participants were obese, 28% were overfat, 20% were healthy, and 2% were under-fat. In month 1 interval, 44% were obese, 34% were overfat, 20% were healthy, and 2% were overfat. In month 2, 36% were overfat, 34% were healthy, 28% were obese, and 2% were under-fat. In month 3, 56% of participants were healthy, 36% were overfat, 6% were obese, and 2% were under-fat.

Lifestyle behaviors of *adults in Lahore*

About 92% of the participants did not exercise regularly, 6% exercised regularly, and 2% reported exercising rarely. 64% of the participants slept five to seven hours, 22% were sleeping more than seven hours, and 14% of the participants were sleeping less than five hours. 58% of the participants reported being moderately active, 38% had a sedentary lifestyle, and 4% of the participants were very active. 94% of the participants showed a willingness to lose weight, while 6% of the participants showed a negative response to willingness to lose weight (Table 3).

**Table 3:** Lifestyle behaviors of adults

Variable	n (%)
How much time is spent in front of a mobile screen	
<2 hours	8 (16)
2-3 hrs	11 (22)
4 hours or more	31 (62)
Exercise level of participants	
Yes	3 (6)
No	46 (92)
Seldom	1 (2)
Smoking status	
Yes	5 (10)
No	43 (86)
Sometimes	2 (4)
Sleep status of the participants	
<5 hours	7 (14)
5-7 hours	32 (64)
More than 7 hours	11 (22)
Activity level of the participants	
Sedentary	19 (38)
Moderate active	29 (58)
Very active	2 (4)
Participants eating in response to	
Anger	5 (10)
Sadness	1 (2)
Boredom	4 (8)
Normal	40 (80)
Participants willingness to lose weight	
Yes	47 (94)
No	3 (6)

Dietary assessment of the participants

The study reveals that 56% of the participants skipped meals while 44% did not take large meals. The majority of the participants consumed more than four meals, with 62% taking tea, 30% taking soft drinks, and 2% choosing fresh juices. The majority of the participants preferred sweets and desserts, while 46% consumed meat and 2% consumed dairy products. Fast food consumption varied, with 42% taking it once a month, 34% weekly, and 4% daily. Cooking oil usage varied, with 54% using cooking oil, 28% using ghee, and 18% using other oils. 44% of the participants reported eating out of home monthly (Table 4).

**Table 4:** Dietary assessment of the participants

Variable	n (%)
Meal skipping by the participants	
Yes	28 (56)
No	22 (44)
Largest meal consumed by the participants	
Breakfast	1 (2)
Lunch	17 (34)
Dinner	10 (20)
No large meal intake	22 (44)
How much gap between each meals	
< 2hrs	2 (4)
3-4 hrs.	18 (36)
>4hours	30 (60)
Intake of Snacks (frequency) by the participants	
1 snack	8 (16)
2 snacks	15 (30)
>3 snacks	5 (10)
Seldom	14 (28)
No snacking	8 (16)
Meal frequency by the participants	
4meals	8 (16)
>4meals	41 (82)
5meals	1 (2)
How many times eating out of home	
Every day	9 (18)
Every week	16 (32)
Per month	22 (44)
Never	3 (6)
Drinks used by the participants	
Coffee	3 (6)
Tea	31 (62)
Fizzy soft drinks	15 (30)
Fresh juice	1 (2)
What are the food choices of the participants	
Dairy products	1 (2)
Meat	23 (46)
Sweets and desserts	26 (52)
Fast-food frequency by the participants	
Daily	2 (4)
Weekly	17 (34)
Monthly	21 (42)
Rarely or never	10 (20)
Which cooking oil used by the participants	
Cooking oil	27 (54)
Ghee	14 (28)
Other oil	9 (18)
Dieting experienced by the participants	
Yes	23 (46)
Seldom	24 (48)
Rarely or never	3 (6)

Body mass index (BMI)

The mean BMI was compared at four intervals (baseline, month 1, month 2, and month 3) in the control group and intervention group to assess the effectiveness of the weight loss program among adults. The participants in the intervention group who received treatment (diet plan and physical activity guidelines) with three-month follow-ups successfully lost weight and had a significant reduction in BMI from baseline to month 3. That was more than what was achieved by the participants in the control group, where no intervention was implemented and the participants only received counseling on a balanced diet. They showed inconsistent results regarding the change in the mean BMI (Table 5).

**Table 5:** Body mass index group differences at four intervals * p<0.05

Time	Control group n (25)	Intervention group n (25)	p-value	t-value	Df	Mean difference (Std. error difference)	95% Confidence interval		Std. Error Mean	
							Lower	Upper	Control group	Intervention group
Baseline	32.16 ± 5.59	26.49 ± 1.59	0.000*	4.87	27.9	5.66 (1.16)	3.28	8.05	1.11	0.31
1 month	30.30 ± 4.91	25.90 ± 1.17	0.000*	4.34	26.73	4.39 (1.01)	2.31	6.46	0.98	0.23
2 months	27.94 ± 4.52	24.62 ± .83	0.001*	3.59	25.64	3.31 (.92)	1.41	5.2	0.9	0.16
3 months	26.04 ± 3.87	23.44 ± .58	0.003*	3.31	25.08	2.60 (.78)	0.98	4.21	0.77	0.11

Waist circumference compared at four intervals

The mean waist circumference was compared at four intervals (baseline, month 1, 2, and month 3) in the control group and intervention group to assess the effectiveness of the weight loss program among the participants. In the intervention group, the participants received treatment (diet plan and physical activity guidelines) with complete three-month follow-ups and lost weight. They showed a significant reduction in waist circumference from baseline to the third month, which was more than in the control group, where no intervention was implemented and the participants only received counseling based on a balanced diet. The control group showed inconsistent results in the change in the mean waist circumference (Table 6). 

**Table 6:** Waist circumference (inches) group differences at four intervals * p<0.05

Time	Control group n (25)	Intervention group n (25)	p-value	t-value	Df	Mean difference (Std. error difference)	95% Confidence interval		Std. Error Mean	
							Lower	Upper	Control group	Intervention group
Baseline	39.92 ± 3.82	36.08 ± 3.52	0.000*	3.76	48	3.91 (1.03)	1.82	6	0.76	0.7
1 month	37.53 ± 2.90	35.36 ± 2.84	0.010*	2.67	48	2.17 (.81)	0.53	3.8	0.58	0.56
2 months	35.37 ± 2.42	34.44 ± 2.46	0.185	1.34	48	.93 (.69)	-0.46	2.32	0.48	0.49
3 months	33.74 ± 2.36	33.90 ± 2.36	0.812	-0.23	48	-.16 (-.67)	-1.5	1.18	0.47	0.47

Waist-to-hip ratio compared at four intervals

The mean value of the waist-to-hip ratio was compared at four intervals (baseline, month 1, month 2, and month 3) in the control group and intervention group. Participants in the intervention group who received treatment (diet plan and physical activity guidelines) showed a decrease in the waist-to-hip ratio from baseline to the third month. However, statistical analysis using the independent t-test at the three-month follow-up revealed no significant difference in the waist-to-hip ratio between the intervention and control groups (p = 0.31). While the intervention group demonstrated a reduction in the waist-to-hip ratio, the control group, which only received counseling on a balanced diet, showed inconsistent results. Overall, the difference between the groups decreased over time, and no statistically significant difference was observed by the third month, suggesting that the intervention's impact on waist-to-hip ratio may have diminished or plateaued (Table 7). There was no statistically significant difference between the control group and the intervention group on the waist-to-hip ratio (WHR) at any of the four time-points (baseline, one month, two months, and three months). At all time points, the mean differences between the groups are very small, ranging from -0.01 to -0.02, with negative values indicating that the Intervention group is slightly higher than the control group in WHR. However, these differences are not statistically significant, as the p-values for all comparisons are much greater than 0.05. This suggests that the Intervention did not have a meaningful or statistically significant impact on WHR over the course of the study, and no significant change was observed between the groups in terms of WHR.

**Table 7:** Waist-to-hip ratio (WHR) group differences at four intervals

Time	Control group n (25)	Intervention group n (25)	p-value	t-value	Df	Mean difference (Std. error difference)	95% Confidence interval		Std. Error Mean	
							Lower	Upper	Control group	Intervention group
Baseline	0.88 ± 0.83	0.89 ± .07	0.629	-0.48	48	-.01 (.02)	-0.05	0.03	0.016	0.015
1 month	0.87 ± 0.07	0.88 ± 0.06	0.632	-0.48	48	-.01 (.02)	-0.05	0.03	0.015	0.013
2 months	0.85 ± 0.07	0.87 ± 0.06	0.44	-0.77	48	-.01 (.02)	-0.05	0.02	0.015	0.013
3 months	0.84 ± .08	0.86 ± .06	0.316	-1.01	48	-.02 (.02)	-0.06	0.02	0.016	0.013

Body fat percentage compared at four intervals

The mean body fat percentage was compared at four intervals (baseline, month 1, month 2, and month 3) in the control group and the intervention group. In the intervention group, the participants received treatment (diet plan and physical activity guidelines) with three-month follow-ups and lost significant body fat from the baseline to month 3. This was more than in the control group, where no intervention was implemented and participants received only counseling based on a balanced diet. The control group showed inconsistent results regarding the change in the mean body fat percentage (Table 8). 

**Table 8:** Body fat (%) group differences from baseline to month 3 * p<0.05

Time	Control group n (25)	Intervention group n (25)	p-value	t-value	Df	Mean difference (Std. error difference)	95% Confidence interval		Std. Error Mean	
							Lower	Upper	Control group	Intervention group
Baseline	42.09 ± 13.40	27.64 ± 8.97	0.000*	4.48	48	14.45 (3.22)	7.96	20.93	2.68	1.79
1 month	38.05. ± 2.06	26.80 ± 1.64	0.000*	4.25	48	11.24 (2.64)	5.93	16.56	2.06	1.64
2 months	33.67 ± 7.25	25.57 ± 7.26	0.000*	3.94	48	8.10 (2.05)	3.97	12.42	1.45	1.45
3 months	30.88 ± 5.69	24.79 ± 6.79	0.001*	3.43	48	6.08 (1.77)	2.51	9.65	1.14	1.35

## Discussion

This study aimed to examine the effectiveness of a weight-loss intervention during 3 months of follow-ups among obese adults enrolled in a randomized controlled trial who were assigned into intervention and control groups. The results of the present study indicate that after three months of nutritional educational intervention, there were considerable weight differences between the intervention group and the control group. The experimental group, which received a diet plan and guidelines on physical activity, showed a significant reduction in weight over time (P < 0.05), a significant reduction in waist circumference, waist-to-hip ratio, and body fat percentage with each follow-up, while the control group, which received a single combined counseling session based on a balanced diet, showed inconsistent results.

The results of our study are similar to those of other studies; Salas-Salvadó et al. [16] discovered that after 12 months of intervention, there were substantial weight differences between the experimental group and the control group (experimental group: -3.2 kg and control group: -0.7 kg; p < 0.001). Additionally, they noticed that the members of the experimental group reduced their waist circumference more than those in the control group (experimental group: -3.3 cm vs. control group: -7 cm; p < 0.001). Furthermore, according to the study, the intervention program significantly reduced waist circumference (pre: 113.1 cm and post: 106.3 cm; p < 0.001), BMI (pre: 35.6 kg/m2 and post: 33.5 kg/m2; p <0 .001), and body weight (pre: 102.5 kg and post: 96.3 kg; p < 0.001).

Previous research has found that those who weigh themselves more frequently while trying to lose weight have better weight loss. In our analysis, the process of being evaluated by an impartial investigator also involves communication with an outside party, which can offer implicit accountability and boost motivation. In our study, we found that the control group, which received more counseling or advice, lost more weight, but meta-regression was unable to rule out chance as the origin of this apparent link. A prospective cohort study showed that individuals with obesity tend to lose weight over time [17]**.** There is a possibility that periodic reweighing and brief weight-loss counseling may result in more weight loss [17].

A study by the Look AHEAD research group showed that the intervention group's mean weight gradually increased after the peak weight loss was reached at 1 year; in the control group, which received a less-intensive intervention, weight loss persisted for the whole eight-year observation period [18]. Sex did not significantly affect weight changes. The high fiber content of plant-based meals and low-fat diets lowered body weight by about 1.6 kg (95% CI 2.0 to 1.2), with no noticeable difference between studies using ovo-lacto vegetarian diets and those employing vegan diets. Research indicates that low-fat, plant-based diets may raise postprandial energy expenditure. It appears that plant-based diets can help people lose weight [18].

Weight control is most closely associated with individuals' food habits and dietary behaviors. Those who adhered to the recommended restrictive eating behaviors were more likely to report weight loss. Food consumption and habits influenced body weight and insulin resistance in a study with a 15-year follow-up, and the association between fast-food habits and lifestyle with weight gain was stronger for White adults than for Black adults. In addition, the human body metabolizes nutrients such as proteins, carbohydrates, and lipids to provide energy, and the metabolic rate is dependent on the type of nutrient and energy intake [19].

Daily exercise and stress levels are also important factors associated with being overweight. Activity level and energy intake significantly affect energy balance in the development of obesity. Stress has been reported to induce glucocorticoids, which stimulate food intake, and insulin, which promotes obesity. The effects of stress and emotional factors foster eating behaviors that can lead to obesity [20].

Studies have shown that for optimum results, nutrition and activity plans must be integrated. In addition to dietary modification, physical exercise should be emphasized as a part of a weight-loss strategy because it is crucial to overall health and weight-loss maintenance [21].

According to the results of the current study, a regularly used 12-week web-based health program helped the intervention group's members lose 4.2% and the control group's members lose 1.4% of their body weight. Similarly, the BMI dropped in the intervention and control groups by 4.0% and 1.6%, respectively. In the Lee et al. study, 44 obese subjects took part in a 12-week weight-loss program. Individual and group sessions focusing on food and activity changes were included in the modules. The results of this study showed that over the 12-week intervention phase, weight and serum triglyceride and free fatty acid concentrations in the obese group decreased significantly with the intervention [22].

The kinds of foods eaten may vary depending on dietary needs and lack of food expertise. Through the provision of educational tools, this intervention intended to promote understanding about suitable food kinds and meals. It seems to have had a good effect on some dietary behaviors because the intervention group dramatically increased their intake of fruit, fiber, and fat-free foods throughout the program [23]. Better behavioral weight-loss program adherence, including attendance, is linked to greater weight loss, but it may be difficult for some people to stick with a longer-term program given that half of the participants in this study worked full-time jobs [24].

Research findings showed that the risk of mortality from all causes decreased by 42% with a daily increase of 10 minutes in moderate-vigorous physical activity in studies of American adults at high risk of a cardiovascular event. The participants were urged to consider their regular eating patterns and to look for ways to consume fewer ultra-processed meals while increasing their intake of fresh foods with minimal processing. Participants frequently reported using this tactic, which involved consuming more raw veggies to substitute ultra-processed foods with natural foods. Participants who were considered to be overweight or obese saw modest decreases in weight and BMI [25]. The public should be encouraged to eat fresher or minimally processed foods, including raw vegetables.

This study has several strengths. It is the first trial to assess the effectiveness of weight loss among adults in Lahore, Pakistan. Key strengths also include successive weight loss among obese adults. The limitation of this trial is its short three-month duration. As relapses of weight gain in the course of several months can happen, longer-term follow-up data would help determine whether participants can maintain their weight loss. The lack of detailed information from these participants on the specific lifestyle changes encouraged, namely, dietary changes, increased physical activity, meal planning strategies, self-monitoring, motivation, and group support, is also a limitation. Another limitation is that the sample size was small, which may be associated with greater uncertainty regarding the measured effect. Because of the small sample size, the differences would have to be greater to reach statistical significance; therefore, although there is a real difference, its existence cannot be guaranteed. Second, this small sample size comes from a single city in Pakistan; therefore, caution should be used when generalizing the findings to other areas of Pakistan. Third, the measures were estimated based on self-reported data, which could imply that some participants underestimated their answers to the questionnaires. It is recommended that larger epidemiological studies be conducted to validate the study further. A larger sample size can be used to get a more representative sample. Fruits and vegetables along with a balanced diet should be recommended to the overweight and obese population.

## Conclusions

Our study showed that there was consistent weight reduction and reduction in waist circumference, waist-to-hip ratio, and body fat percentage among the participants in the intervention group of our study. On the other hand, reductions were observed among control group participants in most outcomes, except for the waist-to-hip ratio (WHR), where no significant change was noted. The age group in our study was young adults between the ages of 18 and 40 years, and most of the participants were earning more than PKR 50,000, which puts them in the middle class. In addition, almost all the participants in our study were employed (96%). Therefore, the weight-loss success was seen among the relatively middle class in our study, but how this intervention will work among other socio-economic classes still needs to be seen.

The rising prevalence of obesity seen around the world is unlikely to be reversed by a single measure. To solve this complicated problem, a thorough strategy is required. Adults who are overweight or obese must be able to acquire and sustain lifestyle behaviors, as well as improve their eating habits, in order to successfully treat their condition. The prescription of a low-fat, high-fiber diet and regular physical exercise has the potential to reduce body weight, which is helpful for the prevention and management of weight-related conditions.
